# Statistical computation for heat and mass transfers of water-based nanofluids containing Cu, Al_2_O_3_, and TiO_2_ nanoparticles over a curved surface

**DOI:** 10.1038/s41598-024-57532-x

**Published:** 2024-03-22

**Authors:** Showkat Ahmad Lone, Zehba Raizah, Anwar Saeed, Gabriella Bognár

**Affiliations:** 1 Department of Basic Sciences, College of Science and Theoretical Studies, Saudi Electronic University, (Jeddah-M), 11673 Riyadh, Kingdom of Saudi Arabia; 2 Department of Mathematics, College of Science, Abha, King Khalid University, Abha, Kingdom of Saudi Arabia; 3 Department of Mathematics, Abdul Wali Khan University, Mardan, 23200 Khyber Pakhtunkhwa Pakistan; 4 Institute of Machine and Product Design, University of Miskolc, Miskolc-Egyetemváros, 3515 Hungary

**Keywords:** Nanofluids, MHD, Brownian motion and thermophoresis, Joule heating, Chemical reaction, Thermal convective condition, HAM, Engineering, Mathematics and computing

## Abstract

Nanofluid is a specially crafted fluid comprising a pure fluid with dispersed nanometer-sized particles. Incorporation these nanoparticles into pure fluid results in a fluid with improved thermal properties in comparison of pure fluid. The enhanced properties of nanofluids make them highly sought after, in diverse applications, consisting of coolant of devices, heat exchangers, and thermal solar systems. In this study hybrid nanofluid consisting of copper, alumina and titanium nanoparticles on a curved sheet has investigated with impact of chemical reactivity, magnetic field and Joule heating. The leading equations have converted to normal equations by using appropriate set of variables and has then evaluated by homotopy analysis method. The outcomes are shown through Figures and Tables and are discussed physically. It has revealed in this study that Cu-nanofluid flow has augmented velocity, temperature, and volume fraction distributions than those of Al_2_O_3_-nanofluid and TiO_2_-nanofluid. Also, the Cu-nanofluid flow has higher heat and mass transfer rates than those of Al_2_O_3_-nanofluid and TiO_2_-nanofluid.

## Introduction

Nanofluid is a specialized engineered fluid comprising a base fluid with dispersed nanometer-sized particles. Introducing these nanoparticles in pure fluid enhances its thermal properties compared to the original fluid as first introduced by Choi and Eastman^1^. The enhanced properties of nanofluids make them highly sought after, in diverse applications, consisting of devices coolant, and thermal solar systems^2,3^. This dynamic field continues to evolve as scientists work towards optimizing nanofluid formulations for practical implementation in a range of thermal management scenarios. Heat transfer in nanofluid flows is characterized by the remarkable improvement in thermal conductivity conferred by nanometer-sized particles mixed in pure fluid^4^. Arshad et al.^5^ used hybrid nanofluid flow through two gyrating surfaces with impacts of chemical reactivity and thermally radiated effects. The presence of these nanoparticles significantly enhances convective heat transfer, making nanofluids attractive for applications demanding efficient thermal management. The nanoparticle size plays a pivotal role, with smaller particles generally contributing to more effective heat transfer^6^. Nanofluids are particularly valuable in cooling systems for electronics, where their superior heat dissipation properties help prevent overheating. The temperature dependency of thermal conductivity, along with the influence on thermal boundary layers near surfaces, is carefully considered for optimizing performance. Despite the promising advantages, challenges such as nanoparticle stability and uniform dispersion remain subjects of ongoing research, reflecting the evolving nature of utilizing nanofluids to enhance heat transfer in various applications^7^. Arshad et al.^8^ explored mass and thermal transportations with first order chemically reactive effects on a dual elongating sheet and conducted a comparative analysis for mono-, bi- and tri-nanoparticles dynamics on flow of fluid. Khan et al.^9^ studied irreversibility generation and heat transference for gyrating nanofluid flow on a revolving cylinder and have noticed that velocity panels have deteriorated while thermal panels have escalated with upsurge in nanoparticles volume fraction. Acharya et al.^10^ inspected variations in hydrothermal nanofluid flow with impacts of diameter of nanoparticles and nano-layer. Arshad et al.^11^ studied time-based mass and thermal transportation on an infinite permeable sheet with impacts of chemical reaction and noticed that growth in Prandtl number has augmented Sherwood and Nussel numbers. Hassan et al.^12^ studied numerically the nanofluid flow through two permeable rotary surfaces and noticed that thermal panels have amplified with escalation in nanoparticles number. Arshad et al.^13^ used magnetic effects in inclined direction on radiative and chemically reactive nanofluid flow on bi-elongating surface. Hussain et al.^14^ modeled mixed convective and visco-elastic nanofluid flow on a circular cylinder with implications of thermal radiations. Nimmy et al.^15^ used solar radiations on thermal performance on a wetted fin and observed that thermal panels have deteriorated with expansion in conductive and convective factor as well as with improvement in thermally conductive factor. Khan et al.^16^ used the concept of Arrhenius activated energy to study unsteady viscous nanomaterial flow with impacts of bio-convection and slip (partial) constraints.

Magnetohydrodynamic (MHD) fluid flow is a field of physics and engineering that investigates the performance of electrically conducting fluids, such as plasmas and liquid metals, in the existence of magnetic field effects^17^. The fundamental principles of electromagnetism and fluid dynamics are combined into a set of equations, known as the magnetohydrodynamic equations, which describe the complex relationship between fluid motion and magnetic forces. In MHD fluid flow, the Lorentz force emerges from the collaboration amid the magnetic field and charged particles in the fluid, influencing flow patterns, turbulence, and instabilities^18^. This field finds applications in diverse areas, including astrophysics, geophysics, and engineering, with relevance to phenomena such as solar winds, magnetic confinement fusion, and the Earth's magnetic field. MHD fluid flow is essential in the study of technological applications like MHD generators, pumps, and magnetic confinement fusion devices, where the manipulation of fluid motion and magnetic fields holds promise for power generation and propulsion^19^. Sing et al.^20^ studied the thermal behavior for magnetized nanofluid flow on an asymmetric conduit filed with Darcy-Brinkman permeable medium. Mahabaleshwar et al.^21^ discussed the influences of CNTs on MHD fluid flow on a contracting/extending sheet subject to heat sink/source and thermal radiations. MHD effects can enhance heat transfer, depending on factors such as the strength and orientation of the magnetic field, fluid conductivity, and the nature of the heat source^22^. MHD fluid flow has many applications like in plasma physics, astrophysics, and magnetic confinement fusion, where heat transfer plays a critical role in achieving and maintaining controlled conditions for sustained energy production through nuclear fusion reactions^23^. Hussain et al.^24^ computed numerically the optimization of entropy for convective Darcy–Forchheimer nanofluid flow on a flat vertical surface with irregular heat sink/source. Many such studies can be seen in Refs.^25–29^.

Brownian motion characterizes the unpredictable and erratic movement of particles suspended in a fluid caused by collisions with neighboring molecules. This phenomenon is particularly significant for nanoparticles and colloidal particles. In Brownian motion, particles experience unpredictable shifts in position, a process driven by thermal energy. Thermophoresis, on the other hand, is a phenomenon in fluid dynamics where suspended particles exhibit a directional motion in reaction to a temperature gradient. When a temperature gradient is present, thermophoresis induces particle migration from regions of lower temperature to higher temperature. Both Brownian motion and thermophoresis play critical roles in nanoparticle transport and assembly, impacting various fields, including nanotechnology, colloidal science, and the design of advanced materials with tailored properties^30,31^. Tawade et al.^32^ used these effects (Brownian motion and thermophoresis) for fluid flow on a linearly elongating sheet with impacts of thermal radiations and chemical reactivity. Upreti et al.^33^ examined Brownian motion and thermophoresis effects on 3D nanoparticles fluid flow on a Riga plate subject to gyrotactic microorganisms. Brownian motion and thermophoresis significantly influence heat transfer processes, particularly at the nanoscale^34^. Brownian motion, driven by thermal fluctuations, induces random movements of nanoparticles or colloids suspended in a fluid. This stochastic motion enhances convective heat transfer by promoting particle dispersion and reducing thermal boundary layer thickness. Simultaneously, thermophoresis, responding to temperature gradients, imparts directed motion to particles, contributing to heat transfer through particle migration in the fluid. Both phenomena play crucial roles in nanoscale heat conduction, influencing the effective thermal conductivity of nanofluids^35^. In applications like heat exchangers and nanofluidic devices, understanding and harnessing the relationship between Brownian motion and thermophoresis are essential for optimizing heat transfer efficiency and designing advanced materials with tailored thermal properties^36^.

Joule heating, also known as ohmic heating, is a process where electrical energy is converted into heat when an electric current passes through a conductor with some resistance^37^. The phenomenon is named after James Prescott Joule, who first described it in the mid-nineteenth century. Joule heating is prevalent in various electrical devices, such as resistive heaters, electric stoves, and incandescent light bulbs, where the electrical energy is intentionally converted into heat for practical applications^38^. In some cases, particularly in electronic devices and integrated circuits, controlling and minimizing Joule heating is crucial to prevent overheating and ensure the efficient operation of the system. The impact of Joule heating on heat transfer is significant, particularly in electronic devices and conductive materials^39^. In electronic components, this phenomenon can influence the overall thermal management of the device. The heat produced due to Joule heating can lead to temperature gradients and thermal stresses, affecting the performance and reliability of the system^40^. Efficient heat dissipation strategies become crucial to prevent overheating and potential damage. Additionally, in applications like microelectronics and integrated circuits, understanding and managing Joule heating is essential for designing effective cooling systems, heat sinks, and thermal interfaces to enhance overall heat transfer and prevent thermal-induced failures in electronic components. Otman et al.^41^ discussed mathematically the analysis of mixed convection stagnant point flow on an extending Riga surface using Joule heating impacts. Irfan et al.^42^ inspected thermally on the depiction of mixed convective and radiative nanoparticles flow with Joule heating effects and has proved that thermal transportation has amplified with upsurge in Eckert number. Prakash et al.^43^ discussed radiative and bio-convective nanoparticles flow of fluid on extending bi-directional sheet with impacts of Joule heating, modified diffusions and MHD effects.

Thermal convective conditions in fluid flow describe the dynamic process of heat transfer through the movement of a fluid, influenced by temperature gradients. Natural convection occurs when temperature differences induce buoyancy forces, causing the fluid to circulate spontaneously. Forced convection involves externally induced fluid motion, often using pumps or fans, to enhance heat transfer. Mixed convection combines aspects of both natural and forced convection, prevalent in scenarios where external forces and buoyancy both contribute to fluid motion^44^. These thermal convective conditions are pivotal in diverse applications, including the design of heat exchangers, cooling systems, and various industrial processes. The optimization of thermal convective systems requires a comprehensive understanding of fluid behavior, geometry, and external influences, achieved through computational modeling, experimentation, and theoretical analyses^45^. Hamza et al.^46^ examined MHD time-dependent flow of fluid with convective constraints at the boundary and have noted that the velocity and thermal distributions have augmented with escalation in Biot number. Rashad et al.^47^ inspected Williamson MHD nanofluid flow on a penetrable surface with convective constraints at the boundary and have proved that convective constraints at the boundary and upsurge in thermal radiations has escalated the thermal distribution and declined the velocity panels. Baag et al.^48^ studied free convective nanofluid flow on a stretching sheet using the impacts of heat source with heating convective constraints at the boundary. The impact of thermal convective conditions on heat transfer in fluid flow is profound, efficient heat exchange and thermal management in various engineering systems, such as heat exchangers or electronic devices; depend on thermal convective conditions^49^. Prasad et al.^50^ studied nanofluid couple stress flow with convective constraints at the boundary using temperature-based characteristics and impacts of MHD.

In this study hybrid nanofluid consisting of copper, alumina and titanium nanoparticles on a curved surface has investigated with impact of chemical reactivity, magnetic field and Joule heating. The leading equations have converted to normal equations by using appropriate set of variables. The purpose of this analysis is to address the subsequent research questions:

- Among water-based nanofluids comprising of $Cu,\,\,Al_{2} O_{3}$ and $TiO_{2}$, which exhibits more pronounced velocity and temperature distributions due to inherent factors?
- Among water-based nanofluids comprising of $Cu,\,\,Al_{2} O_{3}$ and $TiO_{2}$, which demonstrates higher surface drags and heat transfer rates due to inherent factors?
- Between curved and flat surfaces, which surface type experiences predominant effects in terms of velocity and temperature distributions, surface drags, and heat transfer rates?

## Problem statement

**Figure 1:**
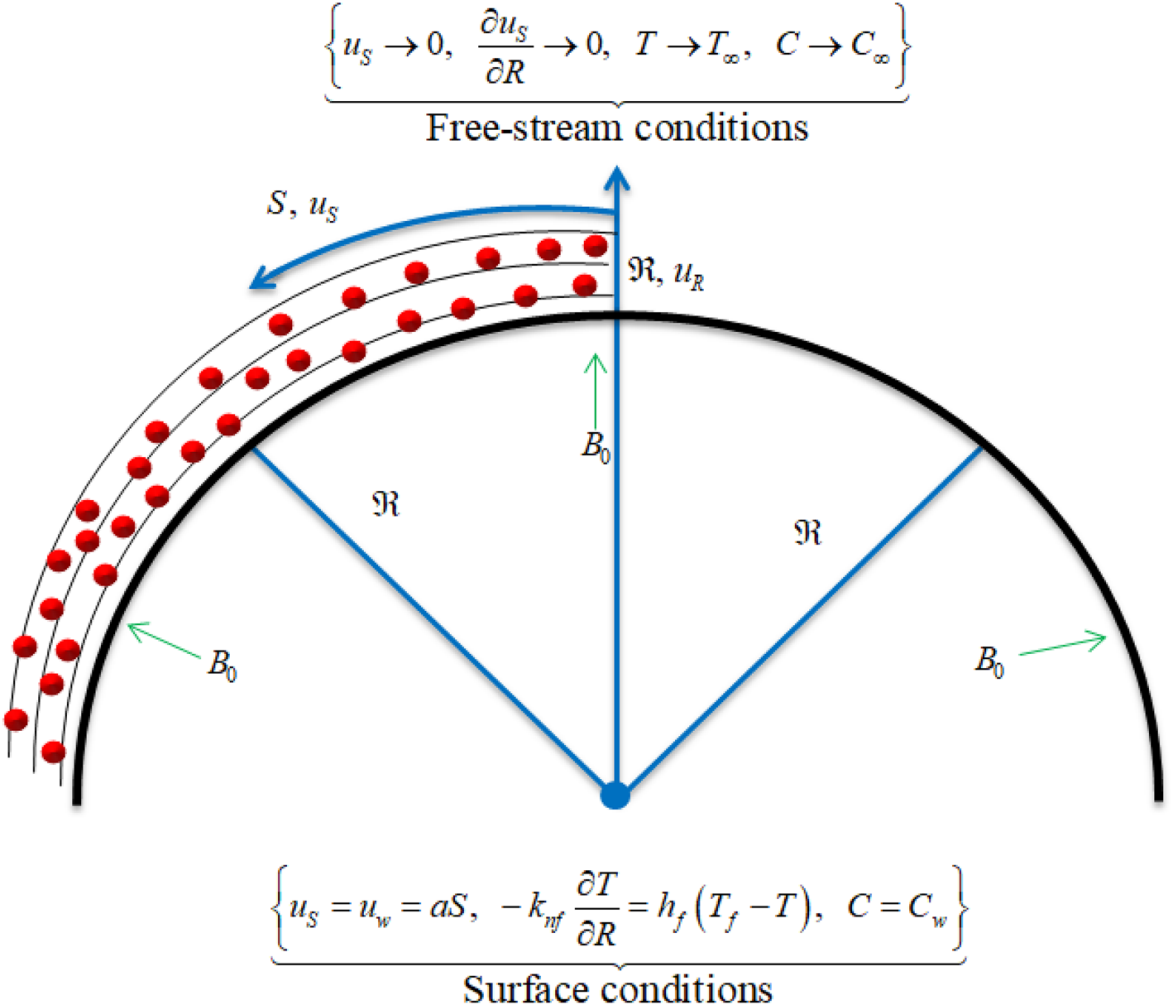
Graphical display of flow problem.

Assume the flow of nanofluids containing copper ($Cu$), alumina (${Al}_{2}{O}_{3}$) and titanium dioxide (${TiO}_{2}$) nanoparticles over a curved surface which is heated up with convective heating. The curved sheet stretches along primary direction, denoted by $S$, with velocity ${u}_{w}=aS$ such that $a>0$. A magnetic field of strength ${B}_{0}$ along $R-$ direction is also taken into consideration. Furthermore, a hot working base fluid (water) having heat transfer coefficient ${h}_{f}$ is taken into consideration. The reference temperature is denoted by ${T}_{f}$ which is greater than the surface temperature ${T}_{w}$ (i.e.,${{T}_{f}>T}_{w}$). Also, the volumetric fraction distribution of the surface is denoted by ${C}_{w}$ and free stream is denoted by ${C}_{\infty }$ as portrayed in Fig. 1. The subsequent conventions have been made to investigate the nanofluids flows:

- The nanofluids flows are affected by the chemical reactivity, Joule heating, and magnetic field.
- The curved surface is taken to be impermeable.
- The curved surface is kept hot with a hot working fluid.

Using above stated assumptions we have^51–53^:

1 $$\frac{{\partial \left( {\left( {R + \Re } \right)u_{R} } \right)}}{\partial R} + \Re \frac{{\partial u_{S} }}{\partial S} = 0,$$

2 $$\frac{{u_{S}^{2} }}{R + \Re } = \frac{1}{{\rho_{nf} }}\frac{\partial p}{{\partial R}},$$

3 $$u_{R} \frac{{\partial u_{S} }}{\partial R} + \frac{\Re }{R + \Re }u_{S} \frac{{\partial u_{S} }}{\partial S} + \frac{{u_{S} u_{R} }}{R + \Re } = - \frac{1}{{\rho_{nf} }}\frac{\Re }{R + \Re }\frac{\partial p}{{\partial S}} + \frac{{\mu_{nf} }}{{\rho_{nf} }}\left( {\frac{{\partial^{2} u_{S} }}{{\partial R^{2} }} + \frac{1}{R + \Re }\frac{{\partial u_{S} }}{\partial R} - \frac{{u_{S} }}{{\left( {R + \Re } \right)^{2} }}} \right) - \frac{{\sigma_{nf} B_{0}^{2} }}{{\rho_{nf} }}u_{S} ,$$

4 $$u_{R} \frac{\partial T}{{\partial R}} + \frac{\Re }{R + \Re }u_{S} \frac{\partial T}{{\partial S}} = \frac{{k_{nf} }}{{\left( {\rho C_{p} } \right)_{nf} }}\left( {\frac{{\partial^{2} T}}{{\partial R^{2} }} + \frac{1}{R + \Re }\frac{\partial T}{{\partial R}}} \right) + \frac{{\sigma_{nf} }}{{\left( {\rho C_{p} } \right)_{nf} }}B_{0}^{2} u_{S}^{2} \, + \frac{{\left( {\rho C_{p} } \right)_{p} }}{{\left( {\rho C_{p} } \right)_{nf} }}\left[ {D_{B} \frac{\partial C}{{\partial R}}\frac{\partial T}{{\partial R}} + \frac{{D_{T} }}{{T_{\infty } }}\left( {\frac{\partial T}{{\partial R}}} \right)^{2} } \right],$$

5 $$u_{R} \frac{\partial C}{{\partial R}} + \frac{\Re }{R + \Re }u_{S} \frac{\partial C}{{\partial S}} = D_{B} \left( {\frac{{\partial^{2} C}}{{\partial R^{2} }} + \frac{1}{R + \Re }\frac{\partial C}{{\partial R}}} \right) + \frac{{D_{T} }}{{T_{\infty } }}\left( {\frac{{\partial^{2} T}}{{\partial R^{2} }} + \frac{1}{R + \Re }\frac{\partial T}{{\partial R}}} \right) - K_{r} \left( {C - C_{\infty } } \right),$$

The constraints at boundaries are^53^:

6 $$\left\{ \begin{gathered} u_{S} = aS = u_{w} ,\,\,\,\,u_{R} = 0,\,\,\,C = C_{w} {,}\,\,\, \, \, - k_{nf} \frac{\partial T}{{\partial R}} = h_{f} \left( {T_{f} - T} \right),\,\,\,\,{\text{ at }}\,R = 0, \hfill \\ \,\,\,\,\,\,\,\,\,\,\,\,u_{S} \to 0,\,\,\,\,\frac{{\partial u_{S} }}{\partial R} \to 0,\,\,C \to C_{\infty } ,\,\,T \to T_{\infty } ,\,\,\,\,{\text{ as }}R \to \infty . \hfill \\ \end{gathered} \right\}$$

The thermophysical features of the nanofluid are:

7 $$\left\{ \begin{gathered} \frac{{\mu_{nf} }}{{\mu_{f} }} = \frac{1}{{\left( {1 - \Pi } \right)^{2.5} }},\,\,\,\,\,\frac{{\rho_{nf} }}{{\rho_{f} }} = \left( {1 - \Pi } \right) + \Pi \frac{{\rho_{s} }}{{\rho_{f} }},\,\,\,\,\,\frac{{\left( {\rho C_{p} } \right)_{nf} }}{{\left( {\rho C_{p} } \right)_{f} }} = \left( {1 - \Pi } \right) + \Pi \frac{{\left( {\rho C_{p} } \right)_{s} }}{{\left( {\rho C_{p} } \right)_{f} }}, \hfill \\ \,\,\,\,\,\,\,\,\frac{{k_{nf} }}{{k_{f} }} = \frac{{k_{s} - 2\Pi \left( {k_{f} - k_{s} } \right) + 2k_{f} }}{{k_{s} + \Pi \left( {k_{f} - k_{s} } \right) + 2k_{f} }},\,\,\,\,\,\frac{{\sigma_{nf} }}{{\sigma_{f} }} = \frac{{\sigma_{s} + 2\sigma_{f} - 2\Pi \left( {\sigma_{f} - \sigma_{s} } \right)}}{{\sigma_{s} + 2\sigma_{f} + \Pi \left( {\sigma_{f} - \sigma_{s} } \right)}}. \hfill \\ \end{gathered} \right\}$$

Table 1 depicts the measured values of pure fluid and nanoparticles.

**Table 1:** Measured values of the base fluid and nanoparticles in the experiment^54–56^.

Properties	$\rho \left[ {\text{J/kg K}} \right]$	$C_{p} \left[ {{\text{kg/m}}^{3} } \right]$	$k\left[ {\text{W/m K}} \right]$	$\sigma \left[ {\text{S/m}} \right]$
$H_{2} O$	997.1	4179	0.613	5.5 × 10^–6^
$TiO_{2}$	4250	686.2	8.9538	2.38 × 10^6^
$Cu$	8933	385	400	1163.10 × 10^7^
$Al_{2} O_{3}$	3970	765	46	131.70 × 10^7^

To convert the equations mentioned earlier, the similarity variables are established as follows:

8 $$\left\{ \begin{gathered} \Gamma = R\sqrt {\frac{a}{{\nu_{f} }}} ,\,\,\,\,u_{S} = aSf^{\prime}\left( \Gamma \right),\,\,\,\,u_{R} = - \frac{\Re }{R + \Re }\sqrt {a\nu_{f} } f\left( \Gamma \right), \hfill \\ p = \rho_{f} \left( {aS} \right)^{2} P\left( \Gamma \right),\,\,\,\,\,\,\varphi \left( \Gamma \right) = \frac{{C - C_{\infty } }}{{C_{w} - C_{\infty } }},\,\,\theta \left( \Gamma \right) = \frac{{T - T_{\infty } }}{{T_{f} - T_{\infty } }} \hfill \\ \end{gathered} \right\},$$

By utilizing the similarity variables mentioned above, Eq. (1) is obvious, and Eq. (2) converted to:

9 $$\frac{{\rho_{f} }}{{\rho_{nf} }}P^{\prime}\left( \Gamma \right) = \frac{1}{\alpha }f^{{\prime}{2}} \left( \Gamma \right),$$

where $\alpha = \Gamma + \delta$. Equation (3) can be reduced as:

10 $$\frac{{\rho_{f} }}{{\rho_{nf} }}\left( {\frac{2\delta }{\alpha }} \right)P\left( \Gamma \right) = \left\{ \begin{gathered} \left( {\frac{{\mu_{nf} /\mu_{f} }}{{\rho_{nf} /\rho_{f} }}} \right)\left( {f^{\prime\prime\prime}\left( \Gamma \right) + f^{\prime\prime}\left( \Gamma \right)\left( {\frac{1}{\alpha }} \right) - f^{\prime}\left( \Gamma \right)\left( {\frac{1}{{\alpha^{2} }}} \right)} \right) - f^{{\prime}{2}} \left( \Gamma \right)\left( {\frac{\delta }{\alpha }} \right) \hfill \\ \,\,\,\,\, - \left( {\frac{{\sigma_{nf} /\sigma_{f} }}{{\rho_{nf} /\rho_{f} }}} \right)f^{\prime}\left( \Gamma \right)M\, + f^{\prime\prime}\left( \Gamma \right)f\left( \Gamma \right)\left( {\frac{\delta }{\alpha }} \right) + f^{\prime}\left( \Gamma \right)\left( {\frac{\delta }{{\alpha^{2} }}} \right)f\left( \Gamma \right) \hfill \\ \end{gathered} \right\}.$$

By taking the derivative of Eq. (10) with respect to $\Gamma$ and is incorporating it into Eq. (9), we derive:

11 $$\begin{gathered} \left( {\frac{{\mu_{nf} /\mu_{f} }}{{\rho_{f} /\rho_{f} }}} \right)\left[ {f^{iv} \left( \Gamma \right) + f^{\prime\prime\prime}\left( \Gamma \right)\left( {\frac{2}{\alpha }} \right) - f^{\prime\prime}\left( \Gamma \right)\left( {\frac{1}{{\alpha^{2} }}} \right) + \left( {\frac{1}{{\alpha^{3} }}} \right)f^{\prime}\left( \Gamma \right)} \right] - \frac{{\sigma_{nf} /\sigma_{f} }}{{\rho_{nf} /\rho_{f} }}M\left[ {f^{\prime}\left( \Gamma \right)\left( {\frac{1}{\alpha }} \right) + f^{\prime\prime}\left( \Gamma \right)} \right] \hfill \\ \,\,\,\,\,\,\, + \left( {\frac{\delta }{\alpha }} \right)\left[ {f^{\prime\prime\prime}\left( \Gamma \right)f\left( \Gamma \right) - f^{\prime\prime}\left( \Gamma \right)f^{\prime}\left( \Gamma \right)} \right] + \left( {\frac{\delta }{{\alpha^{2} }}} \right)\left[ {f^{\prime\prime}\left( \Gamma \right)f\left( \Gamma \right) - f^{{\prime}{2}} \left( \Gamma \right)} \right] - \left( {\frac{\delta }{{\alpha^{3} }}} \right)f^{\prime}\left( \Gamma \right)f\left( \Gamma \right) = 0, \hfill \\ \end{gathered}$$

While rest of the main equations are converted as

12 $$\begin{gathered} \frac{1}{\Pr }\frac{{k_{nf} /k_{f} }}{{\left( {\rho C_{p} } \right)_{nf} /\left( {\rho C_{p} } \right)_{f} }}\left[ {\theta^{\prime\prime}\left( \Gamma \right) + \left( {\frac{1}{\alpha }} \right)\theta^{\prime}\left( \Gamma \right)} \right] + \left( {\frac{\delta }{\alpha }} \right)\theta^{\prime}\left( \Gamma \right)f\left( \Gamma \right) + \hfill \\ Nt\theta^{{\prime}{2}} \left( \Gamma \right)\, + \,Nb\varphi^{\prime}\left( \Gamma \right)\theta^{\prime}\left( \Gamma \right) + \frac{{\sigma_{nf} /\sigma_{f} }}{{\left( {\rho C_{p} } \right)_{nf} /\left( {\rho C_{p} } \right)_{f} }}MEcf^{{\prime}{2}} \left( \Gamma \right) = 0. \hfill \\ \end{gathered}$$

13 $$\phi^{\prime\prime}\left( \Gamma \right) + \left( {\frac{1}{\alpha }} \right)\phi^{\prime}\left( \Gamma \right) + \left( {\frac{Nt}{{Nb\alpha }}} \right)\theta^{\prime}\left( \Gamma \right) + \left( {\frac{\Gamma Sc}{\alpha }} \right)\phi^{\prime}\left( \Gamma \right)f\left( \Gamma \right) + \frac{Nt}{{Nb}}\theta^{\prime\prime}\left( \Gamma \right) - Sc\gamma \phi \left( \Gamma \right) = 0.$$

14 $$\left\{ \begin{gathered} f^{\prime}\left( 0 \right) = 1,\,\,\,\,f\left( 0 \right) = 0,\,\,\,\,\theta^{\prime}\left( 0 \right) = \frac{Bi}{{k_{nf} /k_{f} }}\left( {\theta \left( 0 \right) - 1} \right),\,\,\,\,\phi \left( 0 \right) = 1, \hfill \\ \,\,\,\,\,\,\,f^{\prime}\left( \infty \right) \to 0,\,\,\,\,f^{\prime\prime}\left( \infty \right) \to 0,\,\,\,\,\,\theta \left( \infty \right) \to 0,\,\,\,\,\,\phi \left( \infty \right) \to 0. \hfill \\ \end{gathered} \right\}$$

Pressure can be reduced as:

15 $$P\left( \Gamma \right) = \left( {\frac{\alpha }{2\delta }} \right)\left[ \begin{gathered} \,\,\,\,\,\,\,\frac{{\mu_{nf} }}{{\mu_{f} }}\left( {f^{\prime\prime\prime}\left( \Gamma \right) + \left( {\frac{1}{\alpha }} \right)f^{\prime\prime}\left( \Gamma \right) - \left( {\frac{1}{{\alpha^{2} }}} \right)f^{\prime}\left( \Gamma \right)} \right) - \left( {\frac{{\sigma_{TH} }}{{\sigma_{f} }}} \right)f^{\prime}\left( \Gamma \right)M + \hfill \\ \frac{{\rho_{nf} }}{{\rho_{f} }}\left( {\frac{\delta }{\alpha }} \right)f^{\prime\prime}\left( \Gamma \right)f\left( \Gamma \right) + \frac{{\rho_{nf} }}{{\rho_{f} }}\left( {\frac{\delta }{{\alpha^{2} }}} \right)f^{\prime}\left( \Gamma \right)f\left( \Gamma \right) - \left( {\frac{\delta }{\alpha }} \right)\left( {\frac{{\rho_{nf} }}{{\rho_{f} }}} \right)f^{{\prime}{2}} \left( \Gamma \right). \hfill \\ \end{gathered} \right]$$

16 $$\left\{ \begin{gathered} \,\,\,\delta = \sqrt {\frac{a}{{\nu_{f} }}} \Re ,\,\,\,\,M = \frac{{\sigma_{f} B_{0}^{2} }}{{\rho_{f} a}},\,\,\,\,Ec = \frac{{u_{S}^{2} }}{{\left( {C_{p} } \right)_{f} \left( {T_{f} - T_{\infty } } \right)}},\,\,\,\,\gamma = \frac{{K_{r} }}{a},\,\,\,\,\,\,\,\Pr = \frac{{\left( {\rho C_{p} } \right)_{f} \nu_{f} }}{{k_{f} }}, \hfill \\ Bi = \frac{{h_{f} }}{{k_{f} }}\sqrt {\frac{{\nu_{f} }}{a}} ,\,\,\,Nb = \frac{{\left( {C_{w} - C_{\infty } } \right)D_{B} \left( {\rho C_{p} } \right)_{p} }}{{\nu_{f} \left( {\rho C_{p} } \right)_{f} }},\,\,\,Nt = \frac{{D_{T} \left( {T_{f} - T_{\infty } } \right)\left( {\rho C_{p} } \right)_{p} }}{{\nu_{f} T_{\infty } \left( {\rho C_{p} } \right)_{f} }},\,\,\,Sc = \frac{{\nu_{f} }}{{D_{B} }}. \hfill \\ \end{gathered} \right\}$$

In Eq. (16), $\delta$ shows the curvature factor, $M$ shows magnetic factor, $Bi$ is thermal Biot number, $Ec$ is Eckert number, $\gamma$ signifies chemically reactive factor, $Nb$ is Brownian motion parameter, $Nt$ presents the thermophoresis factor, $Sc$ designates Schmidt number and $\Pr$ shows Prandtl number.

Main quantities are depicted as:

17 $$C_{f} = \frac{{\tau_{RS} }}{{\rho_{f} S^{2} a^{2} }},\,\,\,Nu_{S} = \frac{{Sq_{w} }}{{k_{f} \left( {T_{f} - T_{\infty } } \right)}},\,\,\,Sh_{S} = - \frac{{Sq_{m} }}{{D_{B} \left( {C_{w} - C_{\infty } } \right)}},$$

where

18 $$\tau_{RS} = \mu_{nf} \left. {\left( {\frac{\partial u}{{\partial R}} - \frac{u}{R + \Re }} \right)} \right|_{R = 0} ,\,\,\,q_{w} = - \left. {k_{nf} \frac{\partial T}{{\partial R}}} \right|_{R = 0} ,\,\,\,q_{m} = - \left. {D_{B} \frac{\partial C}{{\partial R}}} \right|_{R = 0} .$$

Using Eq. (8) in Eq. (17) we have

19 $$\left\{ \begin{gathered} \sqrt {{\text{Re}}_{S} } C_{Ss} = \frac{{\mu_{nf} }}{{\mu_{f} }}\left( {f^{\prime\prime}\left( 0 \right) - \frac{1}{\delta }f^{\prime}\left( 0 \right)} \right), \hfill \\ \,\,\,\,\,\,\,\,\,\,\,\,\,\,\frac{{Nu_{S} }}{{\sqrt {{\text{Re}}_{S} } }} = - \frac{{k_{nf} }}{{k_{f} }}\theta^{\prime}\left( 0 \right),\,\,\, \hfill \\ \,\,\,\,\,\,\,\,\,\,\,\,\,Sh = \frac{{Sh_{S} }}{{\sqrt {{\text{Re}}_{S} } }} = - \varphi^{\prime}\left( 0 \right). \hfill \\ \end{gathered} \right\}$$

where ${\text{Re}}_{S} = \frac{{aS^{2} }}{{\nu_{f} }}$ is local Reynolds number.

### Solution by HAM

For the application of HAM, we introduced the initial guesses and linear operators as follows:

20 $$f_{0} \left( \Gamma \right) = 1 - e^{ - \Gamma } ,\,\,\,\,\theta_{0} \left( \Gamma \right) = \frac{Bi}{{\frac{{k_{nf} }}{{k_{f} }} + Bi}}e^{ - \Gamma } ,\,\,\,\,\phi_{0} \left( \Gamma \right) = e^{ - \Gamma } ,$$

21 $$L_{f} \left( \Gamma \right) = f^{iv} - f^{\prime\prime},\,\,\,\,L_{\theta } \left( \Gamma \right) = \theta^{\prime\prime} - \theta ,\,\,\,\,L_{\phi } \left( \Gamma \right) = \phi^{\prime\prime} - \phi ,$$

with properties:

22 $$L_{f} \left( {\sigma_{1} + \sigma_{2} \Gamma + \sigma_{3} e^{ - \Gamma } + \sigma_{4} e^{\Gamma } } \right) = 0,\,\,\,L_{\theta } \left( {\sigma_{5} e^{ - \Gamma } + \sigma_{6} e^{\Gamma } } \right) = 0,\,\,\,L_{\phi } \left( {\sigma_{7} e^{ - \Gamma } + \sigma_{8} e^{\Gamma } } \right) = 0,$$

Above $\sigma_{1} - \sigma_{8}$ are fixed values. For further applications of HAM, please see^57–59^.

### Convergence analysis

**Figure 2:**
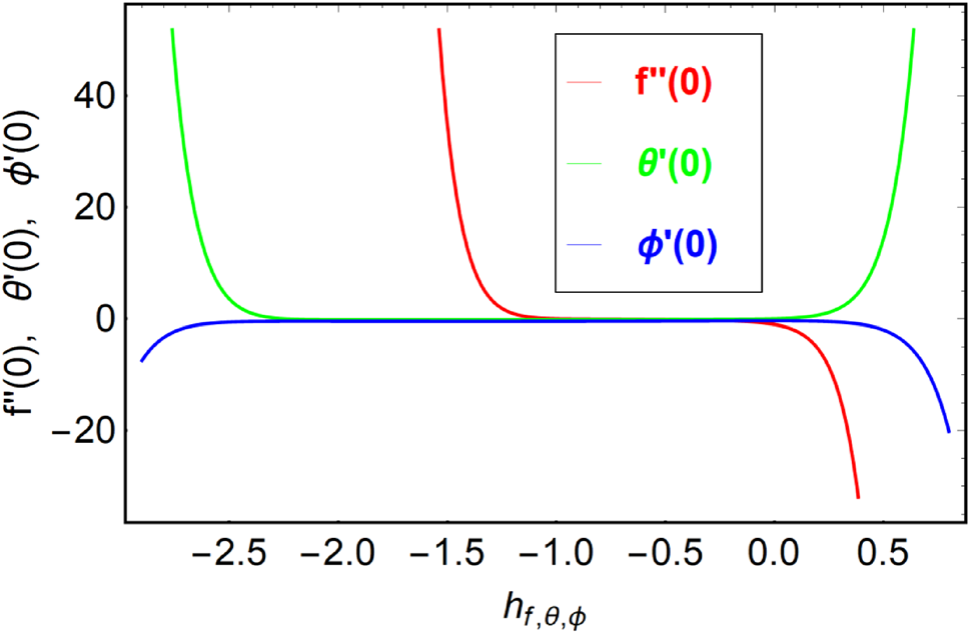
$\hbar -$ curves for $f^{\prime\prime}\left( 0 \right)$, $\theta^{\prime}\left( 0 \right)$ and $\phi^{\prime}\left( 0 \right)$.

This section presents the convergence analysis of the applied method called HAM. This method has a convergence control parameter $\hbar$ which regulates the convergence of HAM. Figure 2 is exhibited to see the convergence area of velocity, temperature and volume fraction distributions. From Fig. 2, we confirm that the convergence of velocity takes place on interval $- 1.0 \le \hbar_{f} \le 0.0$, convergence of thermal panels occur in $- 2.3 \le \hbar_{\theta } \le 0.25$, and volume fraction distribution converges in $- 2.7 \le \hbar_{\phi } \le 0.5$.

### Code confirmation

This section is presented to validate our outcomes with earlier available results. The obtained results are determined for $- \sqrt {{\text{Re}}_{S} } C_{fS}$ while varying $\delta$ and $M = \Pi = 0$. The results are presented in Table 2. From this Table, we have confirmed that the results obtained for a special case are in good relation with those published results.

**Table 2:** Comparison of $- \sqrt {{\text{Re}}_{S} } C_{fS}$ for different values of $\delta$ with $M = \Pi = 0$.

$\delta$	Rosca and Pop^60^	Current outcomes
5	1.15076	1.157565
10	1.07172	1.072534
20	1.03501	1.035464
30	1.02315	1.023463
40	1.01729	1.017346
50	1.01380	1.013995
100	1.00687	1.006963

## Results and discussion

**Figure 3:**
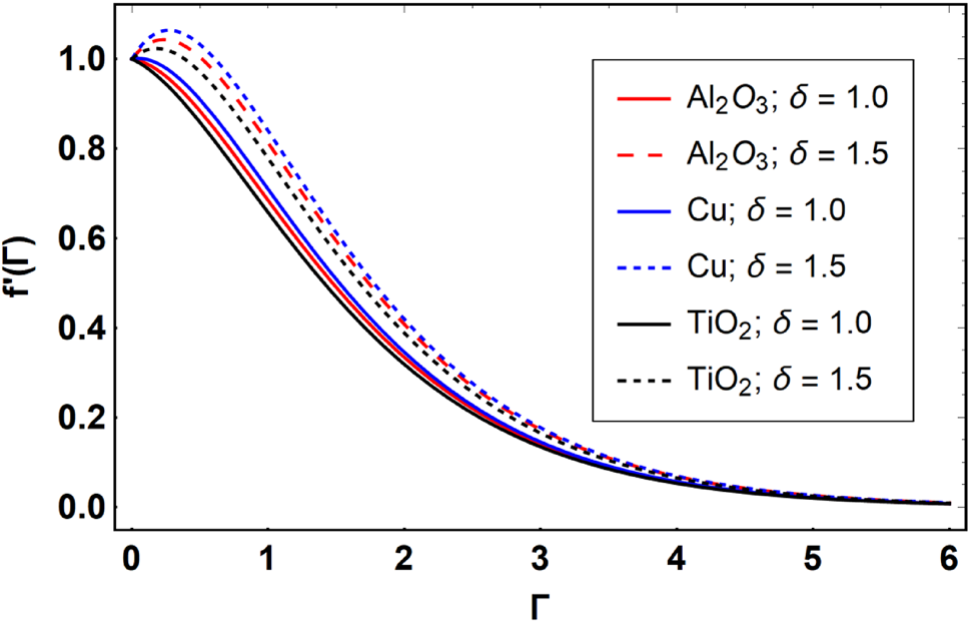
Effect of $\delta$ on $f^{\prime}\left( \Gamma \right)$, when $\Pi = 0.04$, $M = 0.5$, $Ec = 0.1$, $\gamma = 0.5$, $Nb = 0.1$, $Nt = 0.1$, $Sc = 1.0$, $Bi = 0.5$ and $\Pr = 6.2$.

**Figure 4:**
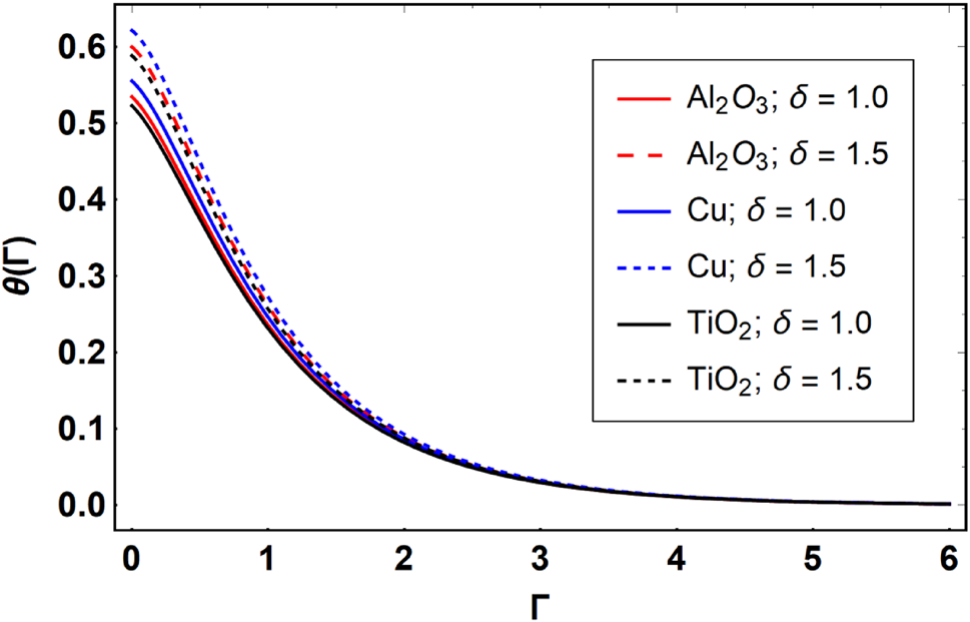
Effect of $\delta$ on $\theta \left( \Gamma \right)$, when $\Pi = 0.04$, $M = 0.5$, $Ec = 0.1$, $\gamma = 0.5$, $Nb = 0.1$, $Nt = 0.1$, $Sc = 1.0$, $Bi = 0.5$ and $\Pr = 6.2$.

**Figure 5:**
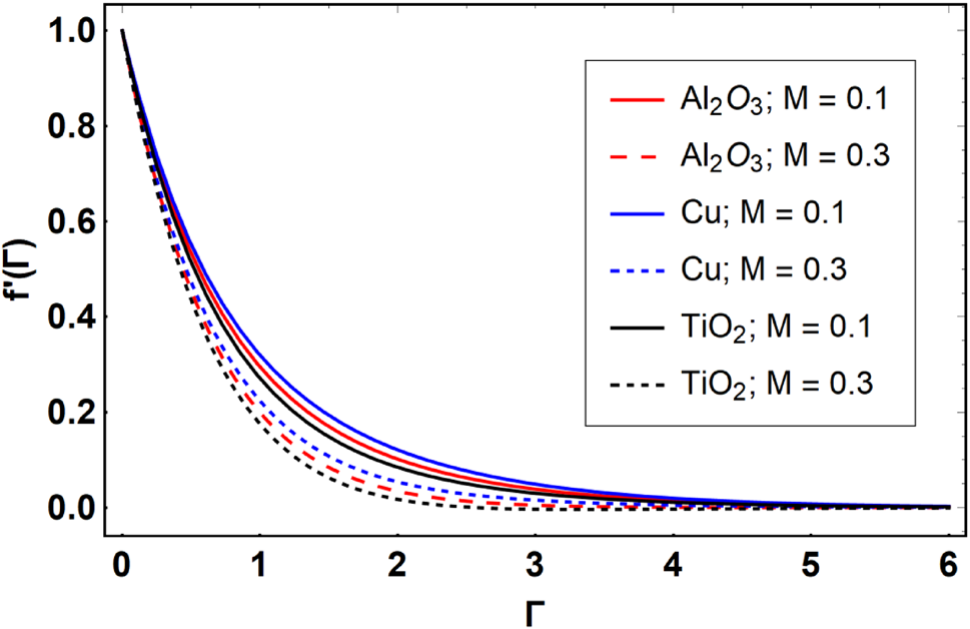
Effect of $M$ on $f^{\prime}\left( \Gamma \right)$, when $\Pi = 0.04$ $\delta = 5.0$, $Ec = 0.1$, $\gamma = 0.5$, $Nb = 0.1$, $Nt = 0.1$, $Sc = 1.0$, $Bi = 0.5$ and $\Pr = 6.2$.

**Figure 6:**
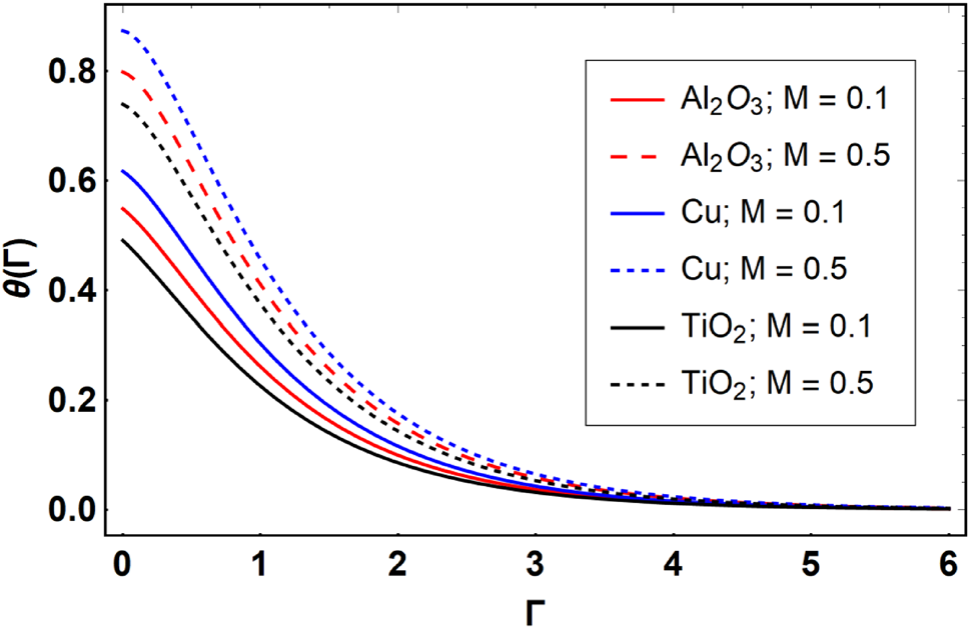
Effect of $M$ on $\theta \left( \Gamma \right)$, when $\Pi = 0.04$ $\delta = 5.0$, $Ec = 0.1$, $\gamma = 0.5$, $Nb = 0.1$, $Nt = 0.1$, $Sc = 1.0$, $Bi = 0.5$ and $\Pr = 6.2$.

**Figure 7:**
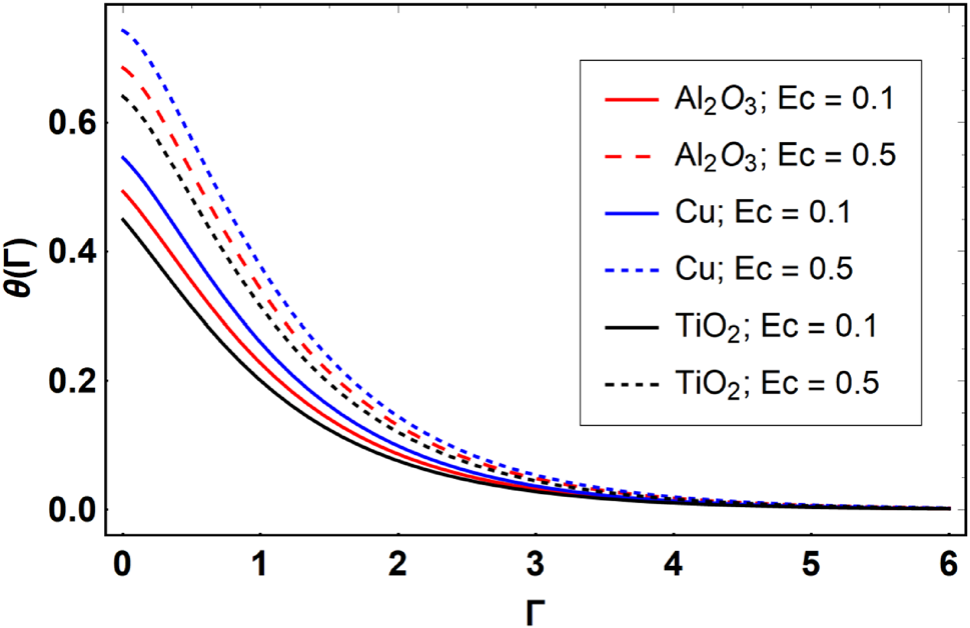
Effect of $Ec$ on $\theta \left( \Gamma \right)$, when $\Pi = 0.04$ $\delta = 5.0$, $M = 0.5$, $\gamma = 0.5$, $Nb = 0.1$, $Nt = 0.1$, $Sc = 1.0$, $Bi = 0.5$ and $\Pr = 6.2$.

**Figure 8:**
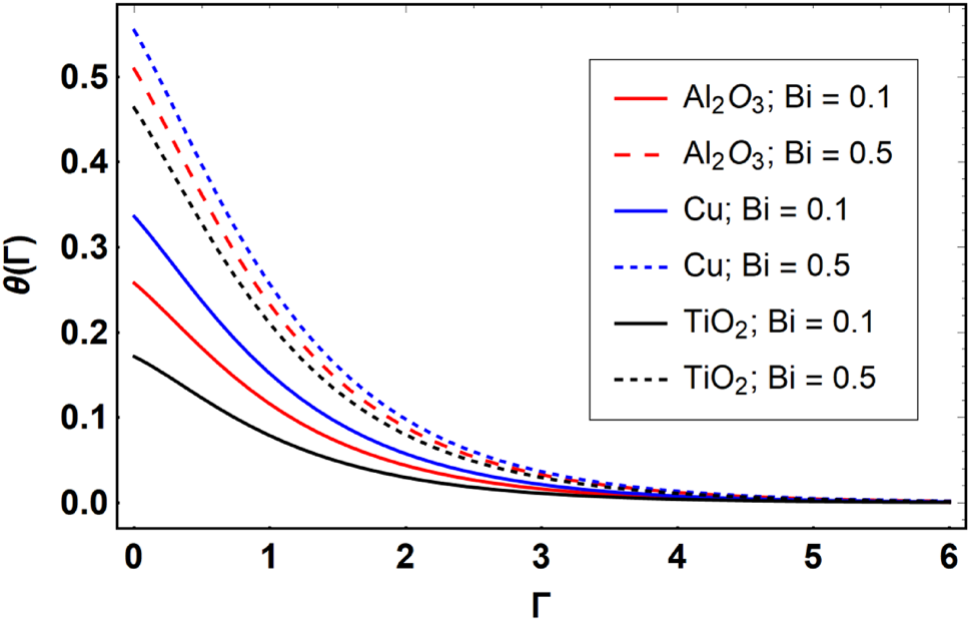
Effect of $Bi$ on $\theta \left( \Gamma \right)$, when $\Pi = 0.04$ $\delta = 5.0$, $M = 0.5$, $Ec = 0.1$, $\gamma = 0.5$, $Nb = 0.1$, $Nt = 0.1$, $Sc = 1.0$ and $\Pr = 6.2$.

**Figure 9:**
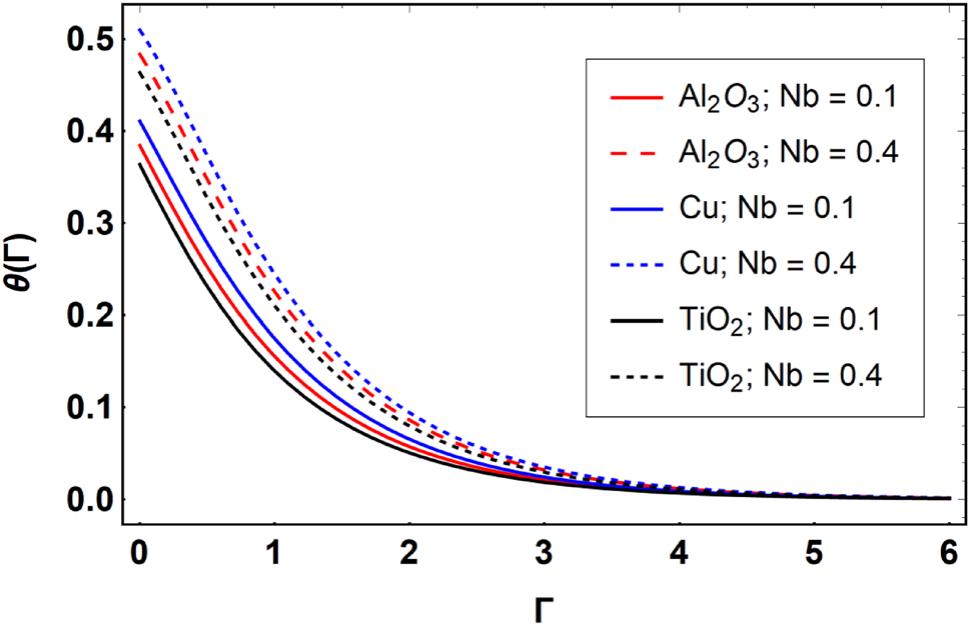
Effect of $Nb$ on $\theta \left( \Gamma \right)$, when $\Pi = 0.04$ $\delta = 5.0$, $M = 0.5$, $Ec = 0.1$, $\gamma = 0.5$, $Nt = 0.1$, $Sc = 1.0$, $Bi = 0.5$ and $\Pr = 6.2$.

**Figure 10:**
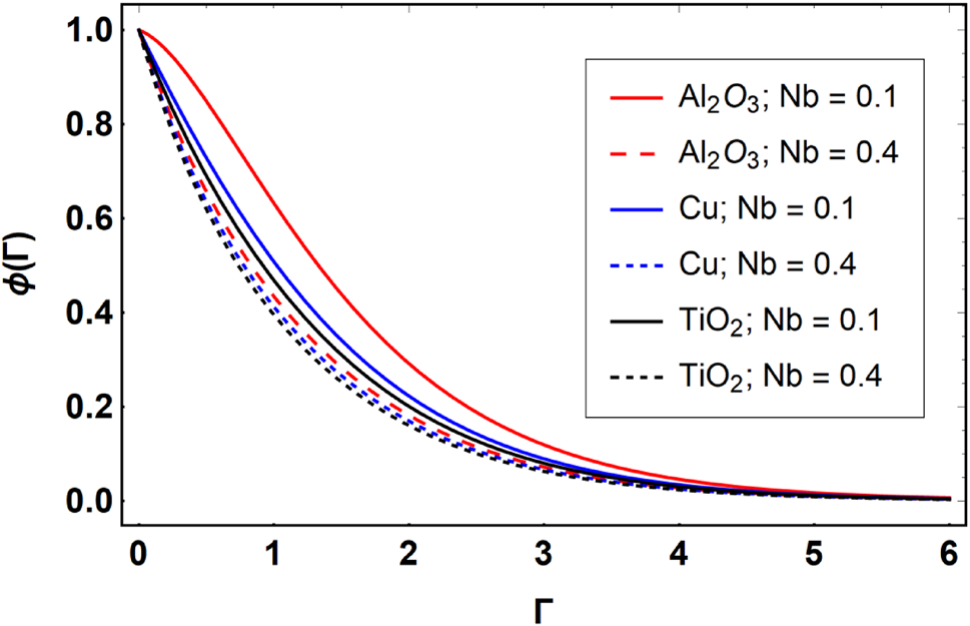
Effect of $Nb$ on $\phi \left( \Gamma \right)$, when $\Pi = 0.04$ $\delta = 5.0$, $M = 0.5$, $Ec = 0.1$, $\gamma = 0.5$, $Nt = 0.1$, $Sc = 1.0$, $Bi = 0.5$ and $\Pr = 6.2$.

**Figure 11:**
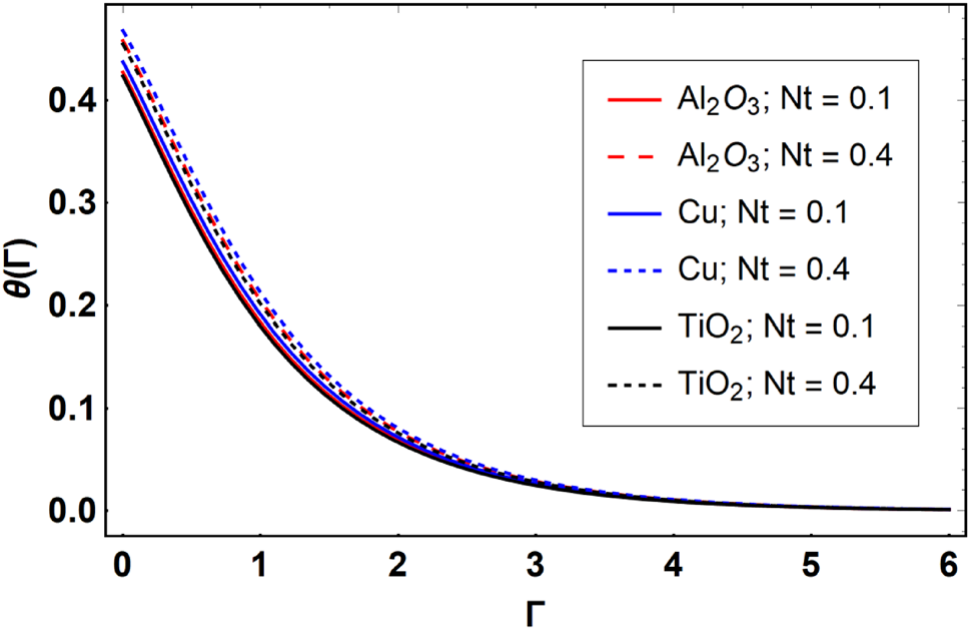
Effect of $Nt$ on $\theta \left( \Gamma \right)$, when $\Pi = 0.04$ $\delta = 5.0$, $M = 0.5$, $Ec = 0.1$, $\gamma = 0.5$, $Nb = 0.1$, $Sc = 1.0$, $Bi = 0.5$ and $\Pr = 6.2$.

**Figure 12:**
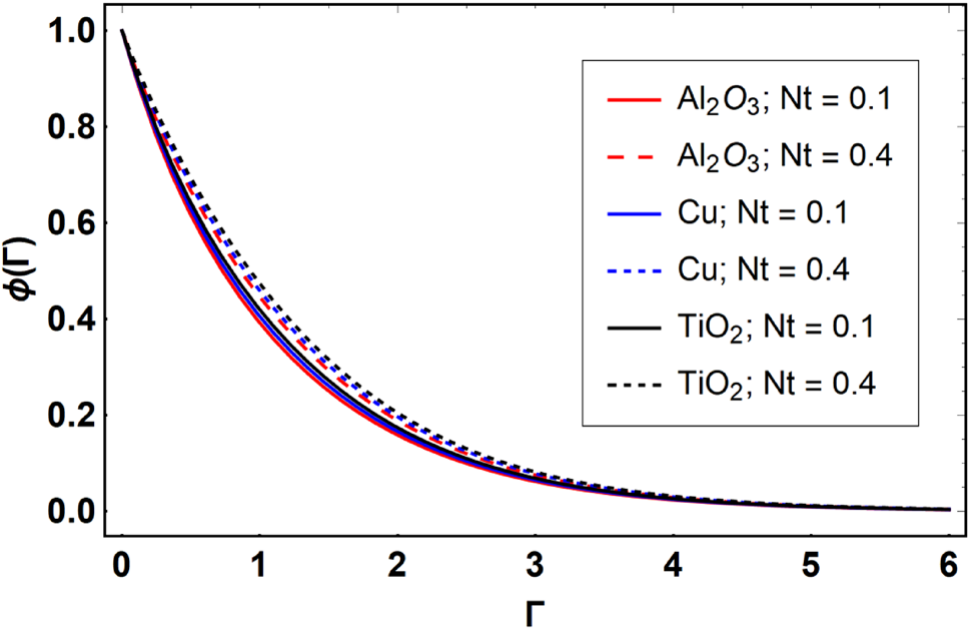
Effect of $Nt$ on $\phi \left( \Gamma \right)$, when $\Pi = 0.04$ $\delta = 5.0$, $M = 0.5$, $Ec = 0.1$, $\gamma = 0.5$, $Nb = 0.1$, $Sc = 1.0$, $Bi = 0.5$ and $\Pr = 6.2$.

**Figure 13:**
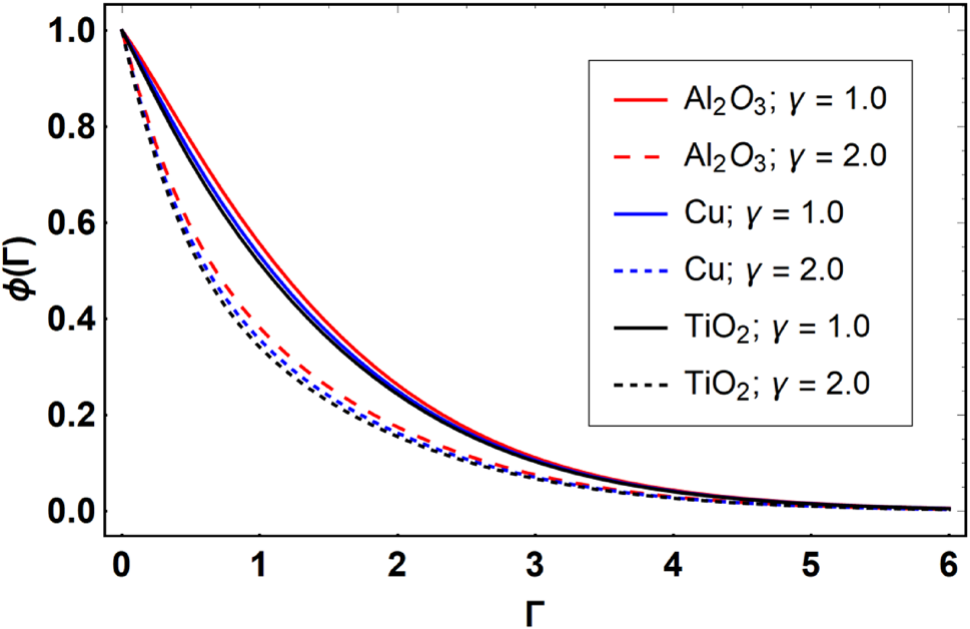
Effect of $\gamma$ on $\phi \left( \Gamma \right)$, when $\Pi = 0.04$ $\delta = 5.0$, $M = 0.5$, $Ec = 0.1$, $Nb = 0.1$, $Nt = 0.1$, $Sc = 1.0$, $Bi = 0.5$ and $\Pr = 6.2$.

**Figure 14:**
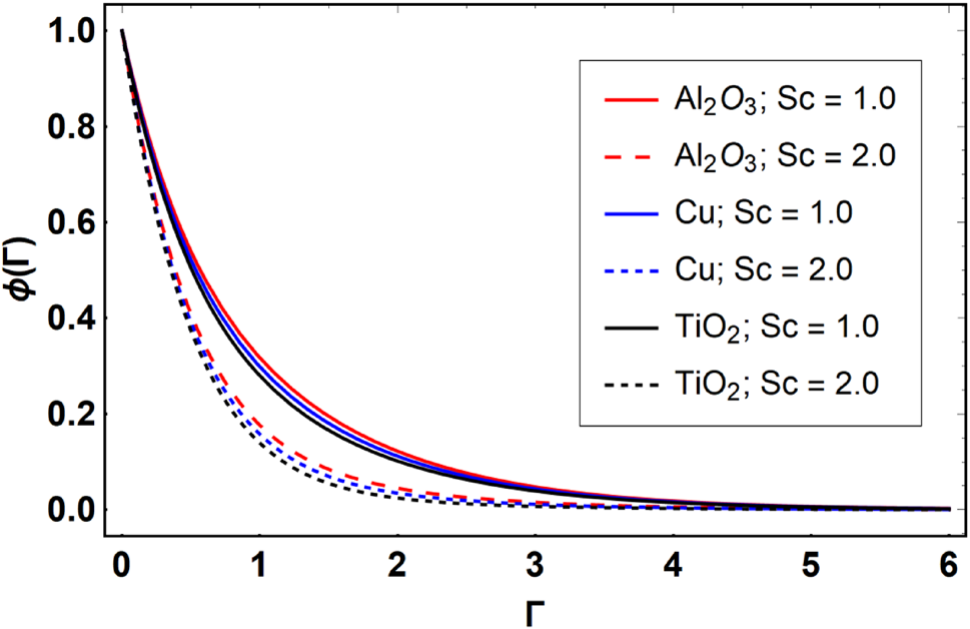
Effect of $Sc$ on $\phi \left( \Gamma \right)$, when $\Pi = 0.04$ $\delta = 5.0$, $M = 0.5$, $Ec = 0.1$, $\gamma = 0.5$, $Nb = 0.1$, $Nt = 0.1$, $Bi = 0.5$ and $\Pr = 6.2$.

This section offers the physical debate of the consequences attained during this analysis. In this study hybrid nanofluid consisting of copper, alumina and titanium nanoparticles on a curved surface has investigated with impact of chemical reactivity, magnetic field and Joule heating. The curved surface is kept hot with a hot working fluid. The curved surface is taken to be impermeable. The HAM method is used to calculate the results for different flow profiles via different embedded factors. The obtained results are exhibited in Figs. 3, 4, 5, 6, 7, 8, 9, 10, 11, 12, 13, and 14, and Tables 3 and 4. The impression of curvature factor ($\delta$) on velocity profiles ($f^{\prime}\left( \Gamma \right)$) of Al_2_O_3_-nanofluid, Cu-nanofluid, and TiO_2_-nanofluid is shown in Fig. 3. Higher $\delta$ increases the velocity profiles of all three types of fluids. The reason is that the increasing $\delta$ means that the curvature of the surface readuces or increases the radius of curvature which makes the surafce flatten. As the surface become flatten then the opposing force at the flatten surface lowers which results higher velocity. The obtained results are investigated in the existence of magnetic field which means that the electrical conductivities of the different nanomaterials (i.e., Al_2_O_3_, Cu, and TiO_2_) has significant role here. From the obtained results, we can see that the Cu-nanofluid has greater velocity than those of Al_2_O_3_ and TiO_2_-nanofluids. The impact $\delta$ on temperature profiles ($\theta \left( \Gamma \right)$) of Al_2_O_3_-nanofluid, Cu-nanofluid, and TiO_2_-nanofluid is portrayed in Fig. 4. An expansion in values of $\delta$ increases the temperature profiles of Al_2_O_3_-nanofluid, Cu-nanofluid, and TiO_2_-nanofluid. Physically, the curvature factor and kinemetic viscosity are inversually proportional to each other which means that the higher curvature factor reduces the kinematic viscosity which results enhancement in $\theta \left( \Gamma \right)$. Hence, the higher $\delta$ increases $\theta \left( \Gamma \right)$. Since from Table 1, we know that the thermal conductivities of the different nanomaterials (i.e., Al_2_O_3_, Cu, and TiO_2_) has significant role here. From the obtained results, we can see that the Cu-nanofluid has greater thermal conductivity than those of Al_2_O_3_ and TiO_2_-nanofluids. Therefore, Cu-nanofluid has greater thermal distribution than those of Al_2_O_3_ and TiO_2_-nanofluids. The impact of $M$ on $f^{\prime}\left( \Gamma \right)$ for Al_2_O_3_-nanofluid, Cu-nanofluid, and TiO_2_-nanofluid is depicted in Fig. 5. The greater values of $M$ reduces the velocity profiles of Al_2_O_3_-nanofluid, Cu-nanofluid, and TiO_2_-nanofluid. Physically, when we increase the magnetic factor, a Lorentz force is created which opposes the motion of fliud particles. This opposing force increases for growth in skin friction at the sheet surface and reduced velocity distribution. The electrical conductivities of the different nanomaterials (i.e., Al_2_O_3_, Cu, and TiO_2_) has significant role here. From the obtained results, we can see that the Cu-nanofluid has greater electrical conductivity than those of Al_2_O_3_ and TiO_2_-nanofluids. Therefore, the Cu-nanofluid has greater velocity than those of Al_2_O_3_ and TiO_2_-nanofluids. The impact of $M$ on $\theta \left( \Gamma \right)$ of the Al_2_O_3_-nanofluid, Cu-nanofluid, and TiO_2_-nanofluid is exposed in Fig. 6. The greater values of $M$ increases the temperature profiles of Al_2_O_3_-nanofluid, Cu-nanofluid, and TiO_2_-nanofluid. As the magnetic strength intensifies, the friction force on the sheet surface rises, leading to a higher rate of thermal transference attributed to increased friction. The higher rate of heat transfer increases the temperature profiles of the nanofluids. Since, Cu-nanofluid has greater electrical conductivity so it will have greater resistive force and rate of heat transfer as well. Therefore, Cu-nanofluid has greater thermal distribution than those of Al_2_O_3_ and TiO_2_-nanofluids. The impression $Ec$ on thermal profiles for Al_2_O_3_-nanofluid, Cu-nanofluid, and TiO_2_-nanofluid is shown in Fig. 7. Greater $Ec$ is responsible for upsurge in $\theta \left( \Gamma \right)$ for Al_2_O_3_-nanofluid, Cu-nanofluid and TiO_2_-nanofluid. The higher $Ec$ transmits the kinetic energy into internal heat energy due to viscous forces which results higher trnasfer rate of heat. Therefore, the $Ec$ upsurges the temperature profiles for all types of fluids. Comparing the three different nanofluids (i.e., Al_2_O_3_-nanofluid, Cu-nanofluid, and TiO_2_-nanofluid), the Cu-nanofluid flow has higher temperature than those of Al_2_O_3_-nanofluid and TiO_2_-nanofluid. Actually, Cu nanomaterial has greater thermal conductance than those of Al_2_O_3_ and TiO_2_ nanomaterials. The impression of $Bi$ (Thermal Biot number) on thermal profiles of the Al_2_O_3_-nanofluid, Cu-nanofluid, and TiO_2_-nanofluid is shown in Fig. 8. Growth in $Bi$ increase the thermal profiles of Al_2_O_3_-nanofluid, Cu-nanofluid and TiO_2_-nanofluid. The higher $Bi$ upsurges the coefficient of thermal tranefernce that supports thegrowth in width of thermal layer at boundary. Therefore, the higher $Bi$ increases $\theta \left( \Gamma \right)$ for Al_2_O_3_-nanofluid, Cu-nanofluid and TiO_2_-nanofluid. Comparing the three different nanofluids, we see that Cu-nanofluid flow has higher temperature than those of Al_2_O_3_-nanofluid and TiO_2_-nanofluid. Actually, Cu nanomaterial has better conductance of heat than those of Al_2_O_3_ and TiO_2_ nanomaterials. The impact of $Nb$ (Brownian motion factor) on thermal and volume fraction distributions on all three types of nanofluid is shown in Figs. 9 and 10. Augmentation in $Nb$ supports thermal distributions while reduce the volume fraction distributions of all three types of fluids. The higher $Nb$ increases the width of thermal layer at boundary, which resutls higher temperature distribution. Since Cu nanomaterial has better conductance of heat than those of Al_2_O_3_ and TiO_2_ nanomaterials, so Cu-nanofluid has a greater $\theta \left( \Gamma \right)$. Conversely, higher values of $Nb$ reduce volume fraction distributions of the Al_2_O_3_-nanofluid, Cu-nanofluid, and TiO_2_-nanofluid. Actually, greater $Nb$ diminishes boundary layer thickness of the volume fraction distribution and causes a drop in the respective profiles of the the Al_2_O_3_-nanofluid, Cu-nanofluid, and TiO_2_-nanofluid. Comparing the three different nanofluids we see that Cu-nanofluid flow has higher temperature than those of Al_2_O_3_-nanofluid and TiO_2_-nanofluid. The impression of thermophoretic factor ($Nt$) on thermal and volume fraction panels for all three types of fluids is shown in Figs. 11 and 12. From the Figures, we see that the greater $Nt$ increases the thermal and volume fraction distributions of the Al_2_O_3_-nanofluid, Cu-nanofluid and TiO_2_-nanofluid. Actually, the greater $Nt$ shows that the thermophoretic forces moves the fluid particles from hotter to colder regions which results higher thermal distribution of the nanofluids flows. Also, the greater $Nt$ increases the volume fraction distribution of the nanofluids flows. Comparing the three different nanofluids (i.e., Al_2_O_3_-nanofluid, Cu-nanofluid, and TiO_2_-nanofluid), the Cu-nanofluid flow has higher temperature and volume fraction distributions than those of Al_2_O_3_-nanofluid and TiO_2_-nanofluid. The impression of chemical reactivity factor ($\gamma$) on volume fraction distributions of the Al_2_O_3_-nanofluid, Cu-nanofluid, and TiO_2_-nanofluid is shown in Fig. 13. From this Figure, we see that the greater $\gamma$ reduces the volume fraction distributions for all three kinds of fluids. The greater $\gamma$ reduces the volumetric boundary layer thickness which results a reduced volume fraction distributions of the Al_2_O_3_-nanofluid, Cu-nanofluid and TiO_2_-nanofluid. Comparing the three different nanofluids (i.e., Al_2_O_3_-nanofluid, Cu-nanofluid, and TiO_2_-nanofluid), the Cu-nanofluid flow has volume fraction distributions than those of Al_2_O_3_-nanofluid and TiO_2_-nanofluid. The impact of Schmidt number ($Sc$) on volume fraction distributions of the Al_2_O_3_-nanofluid, Cu-nanofluid, and TiO_2_-nanofluid is shown in Fig. 14. From this Figure, we see that the greater $Sc$ reduces the volume fraction distributions of the Al_2_O_3_-nanofluid, Cu-nanofluid and TiO_2_-nanofluid. The greater $Sc$ reduces the Brownian diffusivity of the nanofluids flows which result the decreasing volumetric boundary layer thickness. Therefore, the reduced volume fraction distributions of the Al_2_O_3_-nanofluid, Cu-nanofluid and TiO_2_-nanofluid are found here. Comparing the three different nanofluids (i.e., Al_2_O_3_-nanofluid, Cu-nanofluid, and TiO_2_-nanofluid), the Cu-nanofluid flow has higher volume fraction distributions than those of Al_2_O_3_-nanofluid and TiO_2_-nanofluid. Table 3 shows the variation in heat transfer rate $\frac{{Nu_{s} }}{{\sqrt {{\text{Re}}_{s} } }}$ of the three different nanofluids (i.e., Al_2_O_3_-nanofluid, Cu-nanofluid, and TiO_2_-nanofluid) via $\Pi$, $M$, $Ec$, $Nb$, $Nt$ and $Bi$. From the obtained results, we have found that $\frac{{Nu_{s} }}{{\sqrt {{\text{Re}}_{s} } }}$ increases via $\Pi$, $M$, $Ec$, $Bi$ and $Nt$ while reduces via $Nb$. Physically, the higher values of $\Pi$ increase the thermal conductivities of the nanofluids which results higher rate of heat transfers. Comparing the three different nanofluids (i.e., Al_2_O_3_-nanofluid, Cu-nanofluid, and TiO_2_-nanofluid), the Cu-nanofluid flow has higher volume fraction distributions than those of Al_2_O_3_-nanofluid and TiO_2_-nanofluid. Table 4 shows the variation in mass transfer rate $\frac{{Nu_{s} }}{{\sqrt {{\text{Re}}_{s} } }}$ of the three different nanofluids (i.e., Al_2_O_3_-nanofluid, Cu-nanofluid, and TiO_2_-nanofluid) via $Nb$, $Nt$ and $Sc$. From the obtained results, we have found that $\frac{{Nu_{s} }}{{\sqrt {{\text{Re}}_{s} } }}$ increases via $Nb$ and $Nt$ while reduces via $Sc$.

**Table 3:** Impacts of $M$, $Ec$, $Nb$, $Nt$, $Bi$ and $\Pi$ on $\frac{{Nu_{S} }}{{\sqrt {{\text{Re}}_{S} } }}$.

$\Pi$	$M$	$Ec$	$Nb$	$Nt$	$Bi$	$\frac{{Nu_{s} }}{{\sqrt {{\text{Re}}_{s} } }}$		
						$Cu$ nanofluid	$Al_{2} O_{3}$ nanofliud	$TiO_{2}$ nanofluid
0.01						1.224355	1.186543	1.153257
0.02						1.234567	1.196756	1.160753
0.03						1.247648	1.205323	1.170853
0.04						1.253268	1.219638	1.189518
	0.1					0.286474	0.254363	0.225432
	0.2					0.297453	0.269538	0.236851
	0.3					0.306426	0.275373	0.240763
		0.3				0.257563	0.244752	0.228653
		0.6				0.264538	0.259642	0.238532
		0.9				0.274326	0.260864	0.249769
			0.1			0.453353	0.425326	0.387537
			0.2			0.432156	0.406537	0.369633
			0.3			0.415653	0.386273	0.346854
				0.1		0.286463	0.257533	0.236747
				0.2		0.297537	0.268942	0.240863
				0.3		0.305356	0.278095	0.258525
					0.1	0.554535	0.458958	0.415784
					0.2	0.594371	0.497446	0.459048
					0.3	0.647964	0.543589	0.490637

**Table 4:** Sherwood number for variations in $Nb$ and $Nt$.

$Nb$	$Nt$	$Sc$	$\frac{{Sh_{s} }}{{\sqrt {{\text{Re}}_{s} } }}$
0.1			1.685457
0.2			1.697428
0.3			1.708637
	0.1		1.635685
	0.2		1.654374
	0.3		1.679075
		0.1	1.457643
		0.2	1.445784
		0.3	1.435895

## Conclusion

In this study hybrid nanofluid consisting of copper, alumina and titanium nanoparticles on a curved sheet has investigated with impact of chemical reactivity, magnetic field and Joule heating. The leading equations have converted to normal equations by using appropriate set of variables and has then evaluated by homotopy analysis method (HAM). The results are shown through Figures and Tables and are discussed physically.

The ultimate results of the current analysis are:

- The velocity and temperature panels augmenting functions of curvature factor.
- Growing values of magnetic factor reduce the velocity profiles while opposite impact is found for the temperature distribution.
- Brownian motion factor, thermophoresis factor and thermal Biot, Eckert numbers have direct relations with the thermal distribution.
- The Brownian motion, chemical reactivity factors, and Schmidt number has inverse relation with the volume fraction distribution.
- The Cu-nanofluid flow has higher velocity, temperature, and volume fraction distributions than those of Al_2_O_3_-nanofluid and TiO_2_-nanofluid.
- The Cu-nanofluid flow has higher heat and mass transference rates than those of Al_2_O_3_-nanofluid and TiO_2_-nanofluid.

## Data Availability

The data that support the findings of this study are available from the corresponding author upon reasonable request.

## List of symbols

$$\left( {R,S} \right)$$: Curvilinear coordinates (m)
$$u_{w} = aS$$: Stretching velocity (m s^−^^1^)
$a$: Positive fixed number (–)
$$T_{f}$$: Surface temperature (K)
$$\rho$$: Density (kg m^−^^3^)
$$\sigma$$: Electrical conductivity (S m^−^^1^)
$C_{p}$: Specific heat capacity (J K^−^^1^ Kg^−^^1^)
$C_{w}$: Surface volumetric fraction distribution (mol m^−^^3^)
$$k$$: Thermal diffusion (W m^−^^1^ K^−^^1^)
$$\delta$$: Curvature factor (–)
_*M*_: Magnetic parameter (–)
_*Ec*_: Eckert number (–)
_*Bi*_: Thermal Biot number (–)
$\gamma$: Chemical reactivity factor (–)
*N*_*b*_*, N*_*t*_: Brownian and thermophoresis factors (–)
_*Sc*_: Schmidt number (–)
